# Stent-in-stent placement of multi-hole metal stents (M2) for malignant hilar obstruction allows re-intervention as easily as with plastic stents

**DOI:** 10.1055/a-2760-9366

**Published:** 2026-01-13

**Authors:** Hirotsugu Maruyama, Tatsuya Kurokawa, Yuji Kawata, Yoshinori Shimamoto, Yuki Ishikawa-Kakiya, Kojiro Tanoue, Yasuhiro Fujiwara

**Affiliations:** 1 12935 Department of Gastroenterology, Graduate School of Medicine, Osaka Metropolitan University, Osaka, Japan

Endoscopic biliary drainage is widely used for malignant hilar biliary obstruction (MHBO), and plastic stents (PSs), including inside PSs, are commonly employed. Their use is supported by reports showing that the patency of inside PSs is non-inferior to that of metal stents (MSs 1 ), while offering the advantage of easy removal. However, numerous studies have demonstrated that MSs provide longer patency than PSs 2 3 . In patients undergoing antitumor therapy, a stent with both long patency and easy removability is ideal. We previously reported a technique for placing multiple removable multi-hole self-expandable metal stents (MHSEMSs) in MHBO 4 5 . Here, we describe a case in which the stent-in-stent (SIS) placement of MHSEMSs enabled sequential removal using the same technique as that of PSs, allowing easy reintervention.

**Fig. 1:**
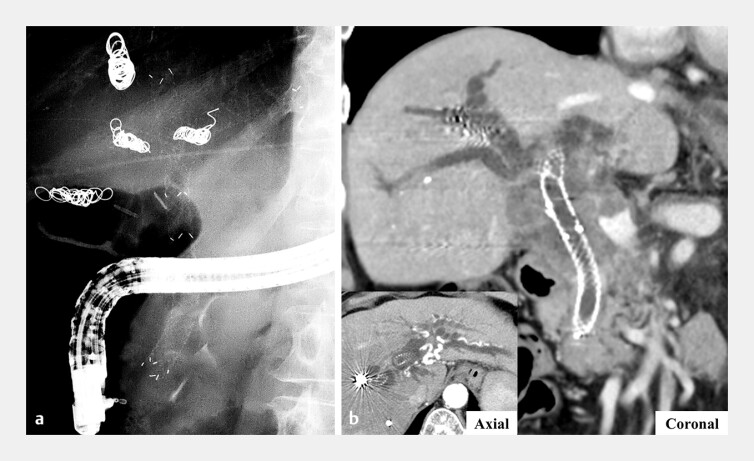
Fluoroscopic and computed tomographic (CT) images. **a** A fluoroscopic image obtained 5 months after the stent-in-stent placement of multi-hole self-expandable metal stents (MHSEMSs). **b** Axial and coronal images showing the dilatation of the bilateral intrahepatic bile ducts.

**Fig. 2:**
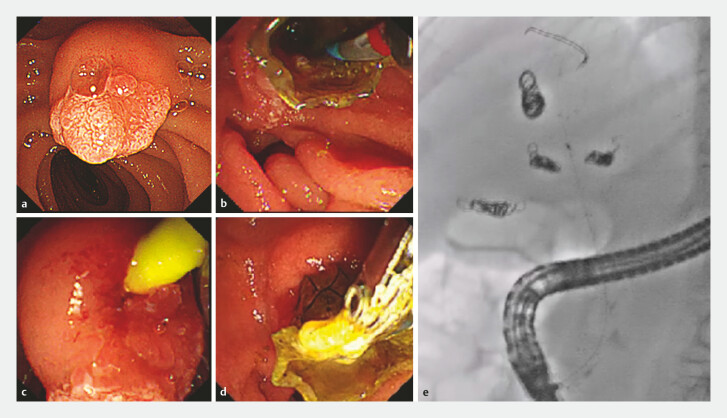
Endoscopic technique for the removal of the MHSEMS. **a** The major papilla was visualized in a frontal view. **b** Biopsy forceps were used to grasp the luminal side of the MHSEMS. **c** The distal end of the MHSEMS was exposed into the duodenum, and a cannula and guidewire (GW) were inserted into the bile duct. **d** A GW was placed in the bile duct, and the MHSEMS was removed using biopsy forceps. **e** A fluoroscopic image with cannula and GW placed inside the MHSEMS.

A 76-year-old woman with hilar bile duct cancer had undergone the SIS placement of the MHSEMS 5 months earlier and was referred for recurrent biliary obstruction ( Fig. 1 ). Using biopsy forceps, each MHSEMS was grasped from the inner lumen under fluoroscopic guidance and gently removed in sequence, similar to inside PS replacement ( Video 1 ). After exposing the distal end of the stent in the duodenum, a 0.025-inch guidewire (GW) was inserted ( Fig. 2 ), facilitating safe removal. The second MHSEMS was then removed in the same manner.

Successful re-intervention as easily as with the plastic stent technique after the stent-in-stent placement of multi-hole metal stents (M2) for malignant hilar obstruction.Video 1

**Fig. 3:**
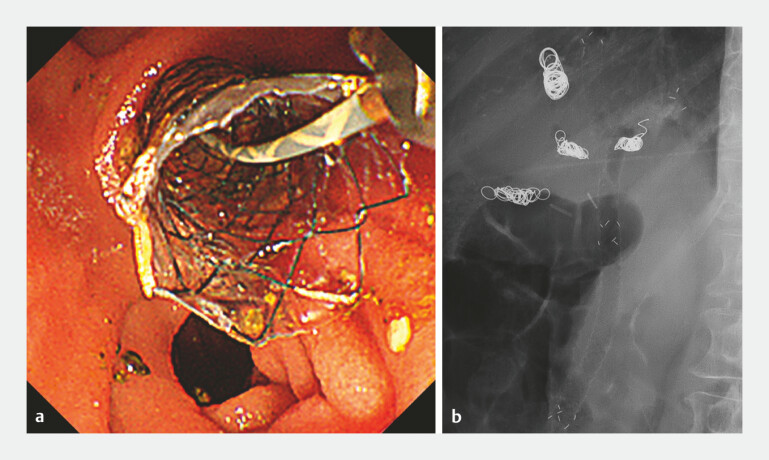
Images of a new MHSEMS placed stent-in-stent. **a** Endoscopic image. **b** Fluoroscopic image.

Following cholangiography to confirm the stenosis, new MHSEMSs were reinserted using the indwelling GW and successfully deployed ( Fig. 3 ). No adverse events occurred.

The SIS placement of the MHSEMS allows each stent to be removed individually, making the procedure as straightforward as with the PS. Moreover, inserting a GW into the larger lumen of an MHSEMS is easier than with a PS. By placing a GW and then removing the stent, the new stent can be securely positioned in the previously drained area, thereby reducing the risk of cholangitis caused by stent misplacement.

Endoscopy_UCTN_Code_TTT_1AR_2AZ
